# Representativeness of an HIV cohort of the sites from which it is recruiting: results from the Ontario HIV Treatment Network (OHTN) cohort study

**DOI:** 10.1186/1471-2288-13-31

**Published:** 2013-03-05

**Authors:** Janet Raboud, DeSheng Su, Ann N Burchell, Sandra Gardner, Sharon Walmsley, Ahmed M Bayoumi, Sandra Blitz, Curtis Cooper, Irving Salit, Jeff Cohen, Sean B Rourke, Mona R Loutfy

**Affiliations:** 1 Toronto General Research Institute, University Health Network, Room 13EN226, 200 Elizabeth St., Toronto, Ontario, M5G 2C4, Canada; 2 Dalla Lana School of Public Health, University of Toronto, Toronto, Ontario, Canada; 3 Ontario HIV Treatment Network, Toronto, Ontario, Canada; 4 Department of Medicine and Institute of Health Policy, Management and Evaluation, University of Toronto, Toronto, Ontario, Canada; 5 Division of General Internal Medicine, Centre for Research on Inner City Health, The Keenan Research Centre in the Li Ka Shing Knowledge Institute, St. Michael’s Hospital, Toronto, Ontario, Canada; 6 The Ottawa Hospital Division of Infectious Diseases, The University of Ottawa, Ottawa, Ontario, Canada; 7 Windsor Regional Hospital, Windsor, Ontario, Canada; 8 Department of Psychiatry, University of Toronto, Toronto, Ontario, Canada; 9 Maple Leaf Medical Clinic, Toronto, Ontario, Canada; 10 Women’s College Research Institute, Women’s College Hospital, Toronto, Ontario, Canada

**Keywords:** Participation bias, Selection bias, HIV, Generalizability, Representativeness

## Abstract

**Background:**

Participation bias is a well-known phenomenon in epidemiologic research, where individuals consenting to research studies differ from individuals who are not able or willing to participate. These dissimilarities may limit the generalizability of results of research studies. Quantification of the participation bias is essential for the interpretation of research findings.

**Methods:**

The Ontario HIV Treatment Network Cohort Study (OCS) is an ongoing open cohort study of HIV positive individuals receiving care at one of 11 sites in Ontario. OCS participants from 4 sites were compared to non-participants (those who declined or were not approached) at those sites with regard to gender, age, HIV risk factor, CD4 count and viral load (VL). Generalized logit regression models were used to identify predictors of declining to participate or not being approached to participate.

**Results:**

Compared to participants (P) in the OCS, individuals who declined to participate (D) and those who were not approached (NA) were slightly younger (D:45, NA:44 vs P:46), less likely to be male (D: 71%, NA:75% vs P:88%), less likely to be Caucasian (D:41%, NA:57% vs P:72%) and less likely to be Canadian-born (D: 39%, NA: 52% vs P: 69%). Patients who were not approached to participate were less likely to have VL < 50 copies/mL than other patients (D: 75%, NA: 62%, P: 74%) and had lower CD4 counts than OCS participants (D: 450 cells/mm^3^, NA: 420 cells/mm^3^, P: 480 cells/mm^3^).

**Conclusions:**

Significant demographic and clinical differences were found between OCS participants and non-participants. Extrapolation of research findings to other populations should be undertaken cautiously.

## Background

Participation bias, also known as volunteer bias and recruitment bias, is a well-known phenomenon in epidemiologic studies where research volunteers differ in important ways from individuals who decline to participate [1-3]. Further, individuals who are not approached to participate in research studies may be even more dissimilar from participants than those who are approached but who decline. Before generalizing the results of a research project to a wider population of interest, it is important to quantify the differences between participants of a research study and individuals from the same population who did not participate.

Population-based studies using non-nominal data from administrative databases or medical charts have been invaluable in HIV research for determining incidence, prevalence and natural history of specific HIV-related conditions [4-8]. While these studies undergo ethics review, individual patient consent is not usually required, so that participation bias does not influence the composition of the study sample.

In contrast, studies of health-related quality of life, mental health, social support and other psychosocial measures require individual patient consent and active participation due to the very nature of the research [9-12]. Although these studies provide important insights into the dynamics and interplay of clinical HIV disease with social factors, the time commitment to complete the questionnaires and willingness to share personal details may yield a study sample which is not representative of the population from which it is drawn.

The Ontario HIV Treatment Network (OHTN) Cohort Study (OCS) [13] is a voluntary clinic-based cohort study of HIV positive individuals living in Ontario, Canada which administers annual interviews to participants. To assess the generalizability of this cohort to HIV positive individuals in care at the recruiting sites, we compared demographic and clinical characteristics of individuals who consented to participate to those of persons from those sites who declined to participate or who were not approached to participate.

## Methods

### Ontario HIV Treatment Network Cohort Study (OCS)

The OCS was designed to collect clinical and socio-behavioural information from people with HIV in Ontario in order to better understand HIV infection, treatment strategies, and their complications; and to improve access to care and treatment for people living with HIV [13]. The OCS was initiated in 2007 and includes both new enrollees as well as persons who had previously participated in the HIV Ontario Observational Database (HOOD) [14] and consented to continue their enrolment.

HIV positive adults were recruited from 11 sites in Ontario, including hospital-based HIV clinics, hospital-based family practice units, and primary care physicians in private practice who specialize in HIV. To be eligible, participants had to have laboratory evidence of HIV infection and be a patient at a currently recruiting site. Approximately 75% of viral load tests conducted at the Ontario Public Health Laboratories, which processes all viral load measurements for clinical care in the province, are submitted by these 11 OCS sites [13]. All study participants sign informed consent prior to participation in the OCS. The OCS has been approved by the University of Toronto Ethics Review Board as well as the ethics review boards of individual sites.

### Data collection

Since September 2007, OCS participants have completed an annual interviewer-administered questionnaire. Two versions of the questionnaire were used: a 20 minute “core” version and a 90–120 minute “extended” version. The core questionnaire collects information on demographic, smoking and alcohol use, health related quality of life, mental health. The 90-minute extended questionnaires collects additional information on psychosocial measures such as social support, coping, stigma, recent life events and ongoing problems. The allocation of questionnaire version to sites was determined based on the preferences of site Principal Investigators and the availability of resources at each site. The core version was administered at seven sites (Maple Leaf Medical Clinic (MLMC) in Toronto, Ottawa Hospital Immunodeficiency Clinic in Ottawa, Windsor Regional Hospital HIV Care Program, HAVEN clinic at the Sudbury Regional Hospital in Sudbury, HIV Clinic in Hotel Dieu Hospital in Kingston, Hamilton Health Sciences’ Chedoke Hospital in Hamilton and St. Joseph’s Hospital in London). The extended version was administered at four sites, all in Toronto (University Health Network (UHN), Sunnybrook Health Sciences Centre and two sites at St Michael’s Hospital). Participants who enrolled at sites administering the core questionnaire were compensated $20 CDN, whereas those who enrolled at sites administering the extended questionnaire were compensated $50 CDN.

At most sites, clinical data were abstracted from medical charts every six months including CD4 counts, and viral load measurements, dates of antiretroviral medications, diagnostic codes, adverse events and hospitalizations. At sites which had established databases for patient care and approval from a local research ethics board, clinical chart data for consenting participants were electronically transferred to the OCS.

### Representativeness study

This representative study was designed to assess the generalizability of participants enrolled in the OCS by comparing them to persons from the clinic sites who declined to participate or who were not approached to participate. All 11 OCS sites were invited to participate in this study and investigators at the following four sites agreed to participate: UHN, MLMC, Ottawa Hospital and Windsor Regional Hospital HIV Care Program. UHN and MLMC are located in Toronto, Canada’s largest city with a population of 2,500,000. UHN is a teaching hospital affiliated with the University of Toronto located in a downtown setting. The Immunodeficiency Clinic at UHN has an active patient population of 1000 comprised largely of men having sex with men (MSM) (48%) and recent immigrants (46% foreign born). MLMC is a community-based clinic providing both primary care and HIV specialty care, with a patient population of approximately 2600, comprised largely of MSM. The Windsor Regional Hospital is located in Essex County, Ontario (population 400,000). The HIV Care Program serves approximately 260 patients with the full spectrum of HIV risk factors. The Ottawa HIV Clinic is located in Ottawa (population 800,000), is affiliated with the University of Ottawa and has an active population of 800, with a significant number of patients infected through injection drug use.

Two types of non-participants were included in the analysis: 1) individuals who declined to participate or withdrew from the study within two weeks whereupon no data was transmitted to the OCS, and 2) individuals who were not approached to participate in the study. The time periods during which participants who consented to participate in the OCS were compared to non-participants were: UHN (February, 2008 – September, 2009) , MLMC (March, 2008 – September, 2009), Ottawa Hospital (February, 2008 – September, 2009) and Windsor Regional Hospital (February 2008 – August, 2009). Patients in HOOD who died or were lost to follow-up and whose data was rolled over into the OCS were not included in this analysis.

Data for the non-participants were obtained from clinic databases for MLMC, UHN and Ottawa and by manual chart review at Windsor Regional Hospital. Each of the three clinic databases contained variables noting the date that each patient was approached to participate in the OCS and whether or not they agreed to participate. Patients who consented were classified as “participants”, patients who were approached but did not agree to participate were classified as “declined” and patients who were not approached during the study period were classified as “not approached”. Patients who were not seen in the clinic during the study period were excluded from the analysis. Additionally, patients at MLMC seen during the study period after the site recruitment quota had been reached were also excluded from the analysis. At Windsor Regional Hospital, a single study nurse was responsible for approaching all patients to participate in the OCS and provided lists of patients who declined to participate and patients who had not been approached. Data for OCS participants were obtained from the OCS database.

This study was approved by the Ethics Review Boards of each of the four participating sites.

### Statistical methods

Demographic and clinical characteristics were compared between participants and non-participants of the OCS. Baseline demographic, clinical and social characteristics were summarized within groups using medians and interquartile ranges (IQR) for continuous variables and frequencies and percentages for categorical variables. For patients who consented, refused to participate or were not approached to participate, baseline values were those at the time of consent, refusal or closest to December 2008 (the midpoint of the study period), respectively.

Characteristics were compared between individuals from the four sites participating in the representativeness study and the other seven OCS sites using chi square tests or Fisher’s exact test as appropriate for categorical variables and Wilcoxon rank sum tests for continuous variables. Characteristics were then compared between participants who consented, individuals who refused and those who were not approached at the four sites participating in the representativeness study using chi square tests or Fisher’s exact test as appropriate for categorical variables and Kruskal-Wallis tests for continuous variables. Multivariable generalized logit regression models were used to identify demographic and clinical characteristics of participants which were associated with the outcomes of declining to participate in the study and not being approached to participate in the study, relative to individuals who consented to participate in the study.

## Results

As of September 2009, 3106 consented participants were enrolled in the OCS, of whom 2925 had completed at least one annual questionnaire. Of these, 1155 participants had completed the extended questionnaire and 1770 participants had completed the core questionnaire. Overall, 85% were male, the median age was 46 (IQR 40–52), 72% were Caucasian, 71% were MSM, 12% were infected with HIV through injection drug use, the median CD4 count was 471 (IQR 330–650) and 73% had viral load < 50 copies/ml at the time of OCS consent, which was a median of 0 days (IQR 0, 59) before the date of completing the first questionnaire (Table 1).

**Table 1:** Comparison of OCS participants’ characteristics between the participating and non-participating sites in the representativeness study

		***All sites***	***4 sites in representativeness study***	***Other sites***	
***Characteristics***		***(n=3106)***	***(n=1861)***	***(n=1245)***	***p value***
Age		46 (40–52)	46 (40–52)	46 (40–52)	0.77
Male		2628 (85%)	1632 (88%)	996 (80%)	<.0001
Risk factor					
	MSM	2133 (71%)	1429 (77%)	704 (60%)	<.0001
	IDU	364 (12%)	180 (10%)	184 (16%)	<.0001
	heterosexual	629 (21%)	325 (18%)	304 (26%)	<.0001
	Blood product/MTCT	197 (6%)	102 (5%)	95 (8%)	0.009
Race					0.02
	Caucasian	2170 (72%)	1329 (72%)	841 (72%)	
	Black/African	418 (14%)	259 (14%)	159 (14%)	
	Aboriginal	161 (5%)	83 (4%)	78 (7%)	
	Other	264 (9%)	176 (10%)	88 (8%)	
Born in Canada		2090 (69%)	1264 (69%)	826 (70%)	0.35
Years since HIV+ test		11 (6–17)	12 (5–18)	11 (6–17)	0.41
Cigarette history					0.006
	Never	1017 (35%)	623 (35%)	394 (35%)	
	Former	736 (25%)	479 (27%)	257 (23%)	
	Current	1161 (40%)	671 (38%)	490 (43%)	
Alcohol use		1102 (38%)	718 (40%)	384 (34%)	0.0003
Substance use		614 (21%)	414 (23%)	200 (18%)	0.0002
Education					<.0001
	High school or lower	929 (32%)	504 (28%)	425 (37%)	
	Some/completed college	890 (30%)	545 (31%)	345 (30%)	
	Some/completed university	1102 (38%)	732 (41%)	370 (32%)	
Employed		1401 (48%)	920 (52%)	481 (42%)	<.0001
Household income					<.0001
	< 30 K	1062 (40%)	566 (35%)	496 (47%)	
	30-60 K	612 (23%)	378 (24%)	234 (22%)	
	60-80 K	298 (11%)	189 (12%)	109 (10%)	
	> 80 K	693 (26%)	470 (29%)	223 (21%)	
General health					<.01
	Good	2353 (81%)	1466 (82%)	887 (78%)	
	Fair	464 (16%)	248 (14%)	216 (19%)	
	Poor	103 (4%)	65 (4%)	38 (3%)	
On cART		2497 (80%)	1487 (80%)	1010 (81%)	0.40
CD4 count (cells/mm^3^)		471 (330–650)	480 (340–650)	460 (310–649)	0.02
VL < 50 copies/mL		2254 (73%)	1379 (74%)	875 (70%)	0.02

*OCS* Ontario HIV Treatment Network Cohort Study, *MSM* men who have sex with men, *IDU* injection drug use, *MTCT* mother to child transmission, *cART* combination antiretroviral therapy, *VL* viral load.

Numbers are reported as median (interquartile range) or frequency (percentage).

Characteristics of patients at the four sites which participated in the representativeness study were compared to those of patients in the seven sites that did not participate (Table 1). Individuals at sites participating in the representativeness study were of similar age, had higher proportions of males and men having sex with men and had higher levels of education and income than individuals at sites that did not agree to participate in the study.

### Comparison of participants vs Non-participants

Baseline characteristics were compared between participants, those invited to participate but who declined and patients who were not approached (Table 2). Participants were similar to non-participants with respect to the proportion who had a history of injection drug use. Participants were slightly older than non-participants, had been infected with HIV longer, had higher CD4 cell counts and were more like to be male, MSM, Caucasian, Canadian-born and be on cART therapy. Individuals who declined to participate had similar rates of virologic suppression at the time they were approached to participate as OCS participants but individuals who were not approached were less likely to have suppressed viral load.

**Table 2:** Comparison of OCS participants to non-participants from four recruiting sites

**Characteristics‡**	**Consented**	**Declined**	**Not approached**	
	**(n =1861)**	**(n =216)**	**(n =502)**	**p value**
Site				
Windsor	128 (7%)	<6 (<3%)	94 (19%)	<.0001
UHN	525 (28%)	162 (75%)	226 (45%)	
Ottawa	293 (16%)	35 (16%)	123 (25%)	
MLMC	915 (49%)	<16 (<7%)	59 (12%)	
Age	46 (40–52)	45 (37–51)	44 (39–50)	0.008
Male	1632 (88%)	154 (71%)	374 (75%)	<.0001
Risk Factor				
MSM	1429 (77%)	90 (42%)	229 (47%)	<.0001
IDU	180 (10%)	13 (6%)	49 (10%)	0.19
Heterosexual	325 (18%)	93 (43%)	177 (36%)	<.0001
Blood product	100 (5%)	10 (5%)	30 (7%)	0.46
Race				
Caucasian	1329 (72%)	76 (41%)	240 (57%)	<.0001
Black/African	259 (14%)	75 (40%)	129 (31%)	
Other	259 (10%)	35 (19%)	49 (11%)	
Born in Canada	1264 (69%)	78 (39%)	224 (52%)	<.0001
Years since HIV+ test	12 (5–18)	7 (5–14)	8 (4–13)	<.0001
On cART	1487 (80%)	156 (73%)	362 (73%)	0.001
CD4 count (cells/mm^3^)	480 (340–650)	450 (296–615)	420 (281–587)	<.0001
<200	155 (8%)	31 (14%)	70 (15%)	<.0001
200-500	851 (46%)	102 (47%)	227 (47%)	
>500	853 (46%)	82 (38%)	185 (38%)	
VL <50 copies/mL	1379 (74%)	160 (75%)	297 (62%)	<.0001

‡ median (interquartile range) or frequency (percent).

*OCS* Ontario HIV Treatment Network Cohort Study, *UHN* University Health Network, *MLMC* Maple Leaf Medical Clinic, *MSM* men who have sex with men, *IDU* injection drug use, *cART* combination antiretroviral therapy, *VL* viral load.

In a multivariable generalized logit regression model (Table 3), MSM (OR = 0.31, p<.0001), patients who were born in Canada (OR = 0.62, p=.01) and patients with a longer time since the first positive HIV test (OR = 0.95 per year, p=.0007) were less likely to decline to participate in the OCS. Individuals with CD4 counts < 200 cells/mm^3^ were twice as likely to decline to participate as individuals with CD4 counts >500 cells/mm^3^ (OR = 2.20, p=.004). Non mutually exclusive risk factors of MSM (OR = 0.30, p<.0001), IDU (OR=0.59, p=.04) and heterosexual contact (OR=0.59, p=.01), a longer time since the first positive HIV test (OR=0.94 per year, p<.0001) and suppressed viral load (OR = 0.59, p=.002) were associated with a lower likelihood of not being approached to participate in the OCS. After adjusting for these covariates, age and gender were not associated with declining to participate in the OCS or not being approached to participate in the OCS. Race was not included in this model due to the unavailability of data on race at one of the sites for non-participants of the OCS.

**Table 3:** Multivariable generalized logit regression model of not participating in the study

***Characteristics***	***OCS participation***	***OR (9% CI)***	***p value***
Site			
UHN (ref.)		1	
MLMC	Declined	0.02 (0.007,0.05)	<.0001
	Not Approached	0.025 (0.01,0.05)	<.0001
OGH	Declined	0.37 (0.24,0.58)	<.0001
	Not Approached	0.80 (0.58,1.09)	0.15
WRH	Declined	0.12 (0.01,0.34)	<.0001
	Not Approached	1.86 (1.31,2.63)	0.0004
Age (per 10 years)	Declined	0.98 (0.82,1.18)	0.86
	Not Approached	0.98 (0.86,1.12)	0.79
Gender			
Female (ref.)		1	
Male	Declined	1.28 (0.81,2.02)	0.29
	Not Approached	1.14 (0.79,1.64)	0.48
Risk factor†			
MSM	Declined	0.31 (0.20,0.45)	<.0001
	Not Approached	0.30 (0.21,0.46)	<.0001
IDU	Declined	0.53 (0.26,1.08)	0.08
	Not Approached	0.59 (0.37,0.97)	0.04
Heterosexual	Declined	0.70 (0.43,1.14)	0.15
	Not Approached	0.59 (0.39,0.89)	0.01
Born in Canada	Declined	0.62 (0.43,0.90)	0.012
	Not Approached	0.87 (0.65,1.16)	0.35
Years since HIV+ test (per year)	Declined	0.95 (0.93,0.98)	0.0007
	Not Approached	0.94 (0.92,0.96)	<.0001
On cART	Declined	0.61 (0.39,0.97)	0.04
	Not Approached	0.91 (0.64,1.31)	0.62
VL <50 copies/mL	Declined	1.28 (0.81,2.02)	0.29
	Not Approached	0.59 (0.42,0.82)	0.002
CD4 count (cells/mm^3^)			
>500 (ref.)		1	
200-500	Declined	1.11 (0.78,1.58)	0.56
	Not Approached	1.02 (0.77,1.33)	0.92
<200	Declined	2.20 (1.29,3.75)	0.004
	Not Approached	1.51 (0.93,2.15)	0.11

*UHN* University Health Network, *MLMC* Maple Leaf Medical Clinic, *MSM* men who have sex with men, *IDU* injection drug use, *cART* combination antiretroviral therapy, *VL* viral load.

† HIV risk factors are not mutually exclusive.

Refusal rates among patients approached to participate in the study varied among sites (2% - 24%), with the highest rate observed at the site which administered the extended questionnaire. The most common reasons site coordinators recalled for declining to participate in the OCS were potential candidates being “too busy”, “not interested” and having concerns about privacy. The most common reasons given for not approaching patients to participate in the OCS included language barriers, perceived fear of disclosure, patient newly diagnosed, mental illness, drug addiction, patient too sick, “difficult” patient and patient was no longer in the target group due to changing recruitment strategies.

## Discussion

Our comparison of participants to non-participants at 4 of the 11 sites of the OHTN Cohort Study (OCS), a volunteer clinic-based study in Ontario, demonstrated that participants were similar to non-participants in terms of the proportions who had a history of injection drug use, and that participants were only slightly older than non-participants. Nevertheless, participants differed from non-participants in a number of important ways. Participants of the OCS were more likely than non-participants to be male, more likely to be Canadian-born, had been infected with HIV longer, had higher CD4 counts, and, compared to those who were not approached to participate, were more likely to have virologic suppression. The latter finding could have been because patients who had not been approached to participate in the OCS during the study period (1) had been diagnosed more recently and had not yet initiated antiretroviral therapy, (2) were further along in their disease and too sick to participate or (3) were less adherent to their treatment. After adjusting for site, the strongest predictors of participation in the OCS in a multivariable model were an HIV risk factor of MSM and a longer duration of HIV infection.

Differences between study participants and non-participants may limit the generalizability of some research findings. For example, the underrepresentation of immigrants in cohort studies may result in the underestimation of the burden of outcomes more common in those populations, such as the prevalence of infection with non-B HIV-1 subtypes, lipohypertrophy (more common among Black women than White women [15]), or social factors unique to newcomers to Canada. Nevertheless, the absence of complete representativeness does not necessarily compromise internal validity for etiologic analyses of risk factors for health outcomes [16]. For example, although individuals with CD4 counts < 200 cells/mm^3^ were less likely to participate, still 83% of those approached did consent, and there is no reason to suspect that their subsequent rate of HIV disease progression, and its predictors, would differ between participants and non-participants.

It should be noted that some clinic patients who had not been approached to participate in the OCS at the time of our analysis may have been approached at a later date, particularly if the reason for not approaching the patient was because a patient was new to the clinic, newly diagnosed, too sick or overwhelmed with other issues at the time, or if the reason the patient was not approached was due to logistical issues within the site regarding time and space for approaching patients.

A variety of considerations factor into the decision of whether or not to participate in a study, including visit frequency, invasiveness of study procedures, travel time required, financial reimbursement, education and the understanding of the importance of research. Language barriers are also a significant issue in this patient population, with a high proportion of recent immigrants. Rates of participation in the OCS may have differed according to whether the site was administering the core questionnaire or the extended questionnaire, since both the time commitment and the financial reimbursement varied by type of questionnaire. It is difficult, however, to obtain accurate and complete information on reasons for which individual patients declined participation or were not approached to participate.

Limited research is available on selection biases in voluntary HIV cohort studies. A study comparing participants, non-participants and dropouts of a cohort of men who have sex with men found significant differences in race, HIV clinical status, income and education, among the three groups but that sexual behaviours were similar [3]. A larger body of work discusses selection biases into intervention studies for HIV positive individuals. Participants in a randomized trial of sexual health research were more likely than non-participants to be women, non-Caucasian, more educated, with more sexual partners and more likely to be returning patients [2]. Not having enough time to participate was the most frequent reason given for not participating. Among patients at a sexually transmitted disease (STD) clinic, attendance at a sexual risk reduction workshop was less likely among younger patients, males, Caucasians and employed individuals but was not related to sexual behaviour or infection status [17]. In an HIV prevention trial among HIV infected individuals, dropouts were more likely to be younger, depressed and not on antiretroviral medications [18]. Also, follow-up was related to how much contact information was collected [19]. Among psychiatric patients in a health promotion study, consenters were at higher risk for drug problems than non-participants and completion of the study was associated with older age, recent STD diagnosis, and a psychiatric diagnosis [20].

Limitations of our study include the fact that a minority of OCS sites agreed to take part in the study and that there was incomplete data regarding reasons for declining to participate or not inviting a patient to participate. Strengths include collection of data from medical charts on individuals who did not participate in the study and classification of these individuals by whether they were invited to participate but declined or were not invited to participate at all.

The OCS has targeted recruitment in recent years in an effort to make the study population more representative of the source population of HIV positive individuals in Ontario seeking medical attention for their HIV disease. The five priority groups for which recruitment is focused are patients who were diagnosed within the past two years, patients younger than 35 years, patients of Aboriginal descent, injection drug users and individuals who were born outside of Canada. While targeted recruitment will increase the numbers of participants in particular subgroups and allow for more detailed study of these individuals, true representativeness is unlikely to be achieved in any voluntary study. There will always be some patients who will be less likely to participate or whom clinic staff is less likely to approach because of issues such as language barriers, advanced disease and mental health problems. It is also important to recognize that attempts to achieve a representative sample must be balanced with a the desire to maximize internal validity in cohort studies by ensuring that participants who do consent are committed volunteers who will be likely to return for follow-up, thereby minimizing selection bias introduced by differential attrition.

## Conclusion

Lack of representativeness does not diminish the significant contribution that a voluntary study with a detailed questionnaire can make to further the understanding of HIV disease on social and clinical outcomes but does require knowledge of the limitations of research with a voluntary cohort. Comparisons with population-based data from administrative databases could also provide important information about the generalizability of results from voluntary cohort studies.

## Competing interests

The authors declare that they have no competing interests related to this paper and project.

## Authors’ contributions

JR, MRL, SW and AMB conceived of the study. DS conducted the statistical analyses under the direction of JMR and MRL. JMR, SB and MRL drafted the manuscript. SR and ANB are Principal Investigator and co-Principal Investigator, respectively, of the OCS and provided insight into recruitment and other issues related to the study CC, JMR, IS, JC and MRL are site investigators of the OCS sites participating in this study. SG contributed insight into recruitment and data management issues of the OCS. All authors reviewed the manuscript during preparation, provided critical feedback and approved the final manuscript.

## Pre-publication history

The pre-publication history for this paper can be accessed here:

http://www.biomedcentral.com/1471-2288/13/31/prepub

## References

[B1] Hagan H McGough JP Thiede H Hopkins SG Weiss NS Alexander ER Volunteer bias in nonrandomized evaluations of the efficacy of needle exchange programs J Urban Health 2000 77 1 103 112 10.1007/BF02350966 10741846 PMC3456613

[B2] Carey MP Senn TE Vanable PA Coury-Doniger P Urban MA Do STD clinic patients who consent to sexual health research differ from those who decline? Findings from a randomized controlled trial with implications for the generalizations of research results Sex Transm Dis 2008 35 1 73 77 10.1097/OLQ.0b013e318148b4ba 18217228 PMC2426775

[B3] Campsmith ML Goldbaum GM Wood RM Demographic and behavioral differences among participants, nonparticipants, and dropouts in a cohort study of men who have sex with men Sex Transm Dis 1995 22 5 312 316 10.1097/00007435-199509000-00008 7502186

[B4] Friis-Moller N Weber R Reiss P Cardiovascular disease risk factors in HIV patients – association with antiretroviral therapy. Results from the DAD Study AIDS 2003 17 1179 1193 10.1097/00002030-200305230-00010 12819520

[B5] Chiesi A Mocroft A Dally LG Regional survival differences across Europe in HIV positive people: The EuroSIDA study AIDS 1999 13 16 2281 2288 10.1097/00002030-199911120-00010 10563713

[B6] Forrest DM Seminari E Hogg RS Yip B Raboud J Lawson L The incidence and spectrum of AIDS-defining illnesses in persons treated with antiretroviral drugs Clin Infect Dis 1998 27 6 1379 1385 10.1086/515030 9868646

[B7] Obel N Engsig FN Rasmussen LD Larsen MV Omland LH Sorensen HT Cohort Profile: The Danish HIV Cohort Study Int J Epidemiol 2009 38 5 1202 1206 10.1093/ije/dyn192 18799495

[B8] Grabar S Selinger-Leneman H Abgrall S Pialoux G Weiss L Costagliola D Prevalence and comparative characteristics of long-term nonprogressors and HIV controller patients in the French Hospital Database on HIV AIDS 2009 23 1163 1169 10.1097/QAD.0b013e32832b44c8 19444075

[B9] Swiss HIV Study C Schoeni-Affolter F Ledergerber B Rickenbach M Cohort Profile: the Swiss HIV Cohort Study Int J Epidemiology 2010 39 5 1179 1189 10.1093/ije/dyp321 19948780

[B10] Kaslow RA Ostrow DG Detels R The Multicenter AIDS Cohort Study: rationale, organization, and selected characteristics of the participants Am J Epidemiol 1987 126 2 310 318 10.1093/aje/126.2.310 3300281

[B11] Justice AC Dombrowski E Conigliaro J Fultz SL Gibson D Madenwald T Goulet J Simberkoff M Butt AA Rimland D Rodriguez-Barradas MC Gibter CL Oursler KAK Brown S Leaf DA Goetz MB Bryant K Veterans Aging Cohort Study (VACS): overview and description Med Care 2006 44 8 Suppl 2 S13 S24 16849964 10.1097/01.mlr.0000223741.02074.66 PMC3049942

[B12] Barkan SE Melnick SL Preston-Martin S The Women’s Interagency HIV Study. WIHS Collaborative Study Group Epidemiology 1998 9 2 117 125 10.1097/00001648-199803000-00004 9504278

[B13] Rourke S Gardner S Burchell AN Raboud JM Rueda S Bayoumi A Loutfy M Cooper C Cohort Profile: The Ontario HIV Treatment Network Cohort Study (OCS) International J Epidemiology 2012 Epub ahead of print 10.1093/ije/dyr230 22345312

[B14] Furler MD Einarson TR Millson M Walmsley S Bendayan R Medicinal and recreational marijuana use by patients infected with HIV AIDS Patient Care STDS 2004 18 4 215 228 10.1089/108729104323038892 15142352

[B15] Andany N Raboud JM Walmsley S Diong C Rourke S Rueda S Rachlis A Wobeser W MacArthur R Binder L Rosenes R Loutfy MR Ethnicity and gender differences in lipodystrophy of HIV-positive individuals taking antiretroviral therapy in Ontario, Canada HIV Clin Trials 2011 12 2 89 103 10.1310/hct1202-89 21498152

[B16] Rothman KJ Greenland S Lash TL Modern Epidemiology 2008 3 Philadelphia: Wolters Kluwer

[B17] Senn TE Carey MP Vanable PA Coury-Doniger P Urban M Even if you build it, we may not come: correlates of non-attendance at a sexual risk reduction workshop for STD clinic patients AIDS Behav 2007 11 6 864 871 10.1007/s10461-007-9218-7 17351737 PMC2416444

[B18] Johnson MO Dilworth SE Neilands TB Chesney MA Rotherman-Borus MJ Remien RH Weinhardt L Ehrhardt AA Morin SF NIMH HLP Team Predictors of attrition among high risk HIV-infected participants enrolled in a multi-site prevention trial. AIDS Behav 2008 12 6 974 977 10.1007/s10461-007-9356-y 18202908 PMC2574761

[B19] Kuhns LM Vazquez R Ramirez-Valles J Researching special populations: retention of Latino gay and bisexual men and transgender persons in longitudinal health research Health Educ Res 2008 23 5 814 825 17974545 10.1093/her/cym066 PMC2574611

[B20] Vanable PA Carey MP Carey KB Maisto SA Predictors of participation and attrition in a health promotion study involving psychiatric outpatients J Consult Clin Psychol 2002 70 2 362 368 11952194 10.1037//0022-006x.70.2.362 PMC2423724

